# Alobar Holoprosencephaly with Cebocephaly in a Neonate Born to an HIV-Positive Mother in Eastern Uganda

**DOI:** 10.1155/2021/7282283

**Published:** 2021-10-25

**Authors:** Franck Katembo Sikakulya, Sonye Magugu Kiyaka, Robert Masereka, Robinson Ssebuufu

**Affiliations:** ^1^Faculty of Clinical Medicine and Dentistry, Department of Surgery, Kampala International University Western Campus, Ishaka-Bushenyi, Kampala, Uganda; ^2^Faculty of Medicine, Université Catholique du Graben, Butembo, Democratic Republic of the Congo; ^3^Department of Surgery, Jinja Regional Referral Hospital, Jinja, Uganda

## Abstract

**Background:**

Holoprosencephaly (HPE) is a rare cerebrofacial abnormality resulting from the complete or partial failure of the diverticulation and cleavage of the primitive forebrain. It has an incidence at birth of 1:16000. *Case Presentation*. We report a case of a 2600 g newborn female delivered by an HIV-infected mother in whom an antenatal ultrasound scan at 34 weeks' gestation reported features of fetal alobar holoprosencephaly. The neonate was born with cebocephaly, a monkey-like head, and did not survive for more than 30 minutes following delivery by caesarian section despite oxygen therapy.

**Conclusion:**

Alobar HPE with cebocephaly remains incompatible with life. In this resource-limited setting, the diagnosis was made clinically, and only an ultrasound scan was performed to confirm the diagnosis. Chromosomal analysis could have given more information.

## 1. Background

Holoprosencephaly (HPE) can be classified into lobar, semilobar, and alobar types and is a rare spectrum of cerebrofacial abnormalities resulting from complete or partial failure of the diverticulation and cleavage of the primitive forebrain [1]. It is associated with midline facial abnormalities such as cyclopia, ethmocephaly, cebocephaly, and premaxillary agenesis [2]. HPE in early embryogenesis has an incidence of 1:250, but at birth, it is around 1:16000, due to a high rate of intrauterine death [3].

TORCH syndrome [3, 4], alcohol and tobacco intake during pregnancy, and diabetes mellitus [5, 6] have been reported to be HPE risk factors. Some cases of HPE have also been reported among HIV-positive mothers [7] with no clear link between maternal HIV status and HPE. Alobar is the most severe form of holoprosencephaly and is incompatible with life [8]. It is mostly associated with cebocephaly which presents with a combination of hypotelorism and a blind-ending single nostril [9]. We report a case of alobar holoprosencephaly with cebocephaly of a neonate born to an HIV-positive mother in a hospital in Eastern Uganda.

## 2. Case Presentation

**Figure 1:**
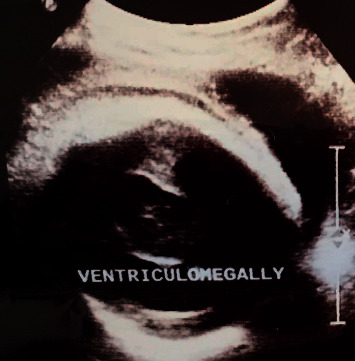
Antenatal ultrasound scan of the fetal head showing a single dilated ventricle with a considerable cortical mantle and a fused thalamus with completely fused hemispheres but without septum pellucidum.

A 2600 g female infant was born to a gravida 3, para 2, 38 year-old HIV-positive female under highly active antiretroviral therapy (HAART) for two years. There was no history of exposure to alcohol, tobacco, teratogens, or ionizing radiation during pregnancy or of any other diseases. An ultrasound scan (USS) performed at 34 weeks of pregnancy reported a single intrauterine fetus in a vertex presentation with a single horseshoe-shaped ventricle, a considerable cortical mantle, and a fused thalamus without septum pellucidum (Figure 1). The other major organs were reported to be of normal appearance.

**Figure 2:**
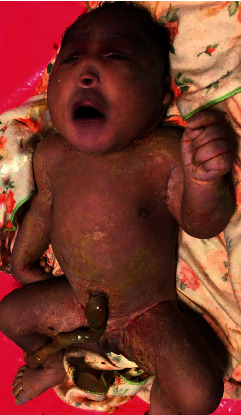
Neonate with cebocephaly (microcephaly, hypotelorism, single nostril, a monkey-like head) and without other external abnormalities.

**Figure 3:**
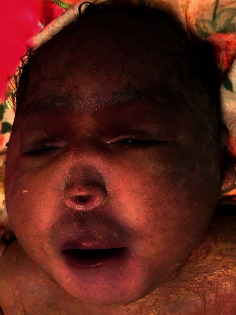
Cebocephaly (monkey-like head).

**Figure 4:**
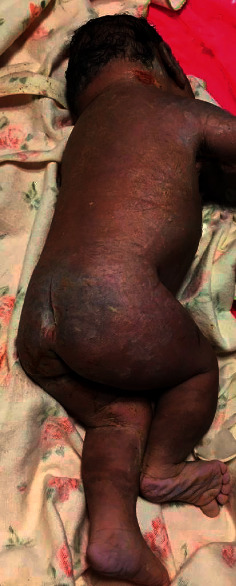
No spina bifida or club foot.

**Figure 5:**
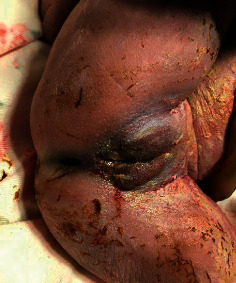
No perineal abnormalities (anus and perineum are normal) in a neonate with alobar HPE.

At 37 weeks, a caesarian section was done due to premature labor and fetal distress, and a female neonate was delivered. She was initially viable with APGAR scores of 6/6/8, at 1/5/10 minutes. Clinically, the neonate had microcephaly (28 cm), hypotelorism, and a single nostril giving a clear picture of a monkey-like head, known as cebocephaly (Figures 2 and 3). There were no other associated external abnormalities such as cleft palate, spina bifida, omphalocele, imperforate anus, or genital abnormalities (Figures 2, 4, and 5). Alobar HPE with cebocephaly diagnosis was made related to the above clinical presentation associated with USS findings showing a single dilated ventricle with a considerable cortical mantle and a fused thalamus with completely fused hemispheres but without septum pellucidum. The neonate was given supplemental oxygen (2 l/minute) due to distress, and 30 minutes later, she passed away due to severe respiratory failure. Any genetic exam or postmortem was not performed.

## 3. Discussion

During the embryological period, primary neurulation is responsible for forming the neural tube. The neural tube forms three important structures: the forebrain, midbrain, and hindbrain [10]. Holoprosencephaly results from incomplete separation of the forebrain into the right and left hemispheres between days 18 and 28 of pregnancy [11].

HPE is categorized into three types: lobar HPE, semilobar HPE, and alobar HPE [1]. In lobar HPE, the brain forms only a partial frontal horn and has an absent corpus callosum and a normal third ventricle; semilobar HPE has partial formation of the interhemispheric fissure and the falx cerebri with complete fusion of the anterior brain, whilst the alobar type has limited formation of the anterior portion of the brain. The infants also lack a falx cerebri, an interhemispheric fissure, and a corpus callosum. In this severe form of HPE, there is no third ventricle, and the thalami are fused [1, 8, 11]. We are presenting a case of alobar HPE with cebocephaly on a neonate born to an HIV-positive mother on HAART for the last two years. The neonate had clinical features of a monkey-like head, and an antenatal ultrasound scan reported a clear picture of alobar HPE with a single ventricle and without a median interhemispheric sulcus.

The etiology of HPE is complex; environmental and genetic factors have been reported [2–4]. Alcohol and tobacco intake during pregnancy, maternal diabetes mellitus, and exposure to TORCH infections, especially cytomegalovirus, have been reported as environmental factors [3–6].

In the pathogenesis of HPE, at least 12 regions on 11 different chromosomes have been implicated [2, 12]. HPE occurs in about 70% of patients with trisomy 13 [12]. This case did not have any association with the abovementioned external environmental factors. Genetic screening was not done to identify whether there was any link with trisomy 13 or any other chromosomal abnormalities. The mother was HIV-positive on HAART and followed up for the last two years. Some cases have been reported among mothers with HIV. However, the link between HPE and maternal HIV status has not been established [7].

Antenatal diagnosis of alobar HPE with cebocephaly by USS has been reported [13]. The USS will show intracranial abnormalities and associated facial malformations indicating a clear antenatal diagnosis of HPE. The intracranial findings include monoventricle and fused thalami and the absence of midline structures, whilst cebocephaly, cyclopia, hypotelorism, and cleft lip are important facial characteristics in the antenatal diagnosis of patients with HPE [1, 8, 9]. In this case, an USS was performed and reported features suggesting holoprosencephaly, but no other investigations were conducted to confirm the findings on USS. It has been reported that fetal MRI can confirm USS findings and can detect additional anomalies. Postnatal MRI with diffusion fiber tractography may detect the rare association of brain stem and long tract abnormalities in holoprosencephaly [14]. The alobar form of holoprosencephaly is incompatible with life, and in our case, the neonate passed away 30 minutes following delivery.

## 4. Conclusion

We present a case of alobar HPE with cebocephaly in an infant born to an HIV-positive mother in Eastern Uganda. It was shown that a well-done antenatal USS can be useful in a resource-limited setting to confirm the diagnosis and inform a decision regarding either termination or progression of the pregnancy. It is important to check for other abnormalities in any neonate with a monkey-like head to rule out HPE. Genetic analysis could help local healthcare workers to identify chromosomal abnormalities and associated phenotypic features. The government should make such investigations available by better equipping laboratories to diagnose genetic disorders.

## Data Availability

No datasets were generated or analysed during the current study.

## Abbreviations

APGAR: Appearance, pulse, grimace, activity, and respiration
HIV: Human immunodeficiency virus
HPE: Holoprosencephaly
TORCH: Toxoplasmosis, other agents, rubella, cytomegalovirus, and herpes simplex
USS: Ultrasound scan.
